# Impact of Chronic Kidney Disease on Risk of Incident Atrial Fibrillation and Subsequent Survival in Medicare Patients

**DOI:** 10.1161/JAHA.112.002097

**Published:** 2012-08-24

**Authors:** Sarah E. Nelson, Gautam R. Shroff, Shuling Li, Charles A. Herzog

**Affiliations:** 1 Department of Medicine, Hennepin County Medical Center and University of Minnesota, Minneapolis, MN (G.R.S., C.A.H.); 2 Division of Cardiology, Department of Medicine, Hennepin County Medical Center and University of Minnesota, Minneapolis, MN (S.E.N.); 3 Cardiovascular Special Studies Center, United States Renal Data System, Minneapolis Medical Research Foundation, Minneapolis, MN (S.L., C.A.H.)

**Keywords:** atrial fibrillation, chronic kidney disease, epidemiology, mortality

## Abstract

**Background:**

Atrial fibrillation (AF) and chronic kidney disease (CKD) are prevalent in the elderly and are independently associated with increased risk of death. We evaluated risk of incident AF with advancing CKD and examined the mortality rate associated with CKD after incident AF in elderly patients.

**Methods and Results:**

This retrospective cohort study used the Medicare 5% database. Point-prevalent Medicare enrollees on December 31, 2006, without preexistent AF or end-stage renal disease were followed up for incident AF through 2008. CKD and AF were identified from *International Classification of Diseases, Ninth Revision, Clinical Modification* diagnosis codes. Associations between CKD stage and incident AF and subsequent risk of death were examined in a Cox proportional-hazards model. Unadjusted survival after incident AF was estimated by the Kaplan-Meier method. CKD was present in 55 962 patients (5.1% of the cohort). Of these, 4952 (8.8%) had CKD stages 1 and 2; 19 795 (35.3%), stages 3 to 5; and 31 215 (55.7%), unknown stage. The hazard ratio for incident AF in CKD stages 3 to 5 was 1.13 (95% confidence interval 1.09 to 1.18). Other stages were not independently associated with incident AF. Survival after incident AF decreased progressively as CKD stage increased (*P*<0.0001). The 1-year mortality rate for CKD stages 3 to 5 with incident AF was 35.6%. Adjusted hazard ratios for death after incident AF were 1.14 (95% confidence interval 1.00 to 1.30) for CKD stages 1 and 2, 1.27 (95% confidence interval 1.20 to 1.35) for CKD stages 3 to 5, and 1.29 (95% confidence interval 1.23 to 1.36) for unknown stage.

**Conclusions:**

Advanced CKD is associated with increased risk of incident AF. In Medicare patients with incident AF, mortality rates are higher for those with advanced CKD than for those without CKD. **(*J Am Heart Assoc*. 2012;1:e002097 doi: 10.1161/JAHA.112.002097.)**

## Introduction

The US subpopulation ≥65 years of age is expected to constitute up to 20% of the total US population by 2050.^1^ Chronic kidney disease (CKD) currently affects nearly 8 million Americans and 8.5% of the Medicare population.^2–3^ Atrial fibrillation (AF) is the most common rhythm abnormality in patients with CKD.^4^ It is estimated that ≍19% to 24% of patients with CKD are diagnosed with AF, and the combined prevalence of these conditions has increased over time. CKD and AF share common risk factors, including preexisting cardiovascular disease and increasing age.^5–6^ Both AF and CKD confer a significant morbidity burden and are independently associated with increased risk of death.^7–8^ AF is associated with a 4- to 5-fold increased risk of stroke^9^ and an ≍2-fold increased risk of death after adjustment for other cardiac conditions.^10^ CKD is independently associated with an increased risk of all-cause death.^6,8^

Few studies have examined CKD as a risk factor for incident AF in elderly patients. Recently, a mortality link was demonstrated between AF and decreasing glomerular filtration rates.^11^ In a large retrospective cohort of Medicare patients, we sought to examine the relationship between advancing CKD stage and incidence of AF and the association between incident AF and subsequent death in patients with CKD. We hypothesized that incidence of AF is higher in patients with advancing CKD stages and that development of AF would be associated with increased risk of death in patients with advancing CKD.

## Methods

### Data Source, Study Population, and Study Period

The Medicare 5% database is a rolling replacement cohort released yearly by the Centers for Medicare and Medicaid Services. It is composed of a random sample of 5% of all Medicare beneficiaries; included patients are followed up until death, and new patients are added yearly.^12^ The study cohort included point-prevalent Medicare enrollees on December 31, 2006, who were enrolled continuously in Medicare with inpatient/outpatient and physician/supplier coverage in 2006; were ≥66 years of age on December 31, 2006; and were residents of the 50 states, the District of Columbia, Puerto Rico, or the Territories. Patients with end-stage renal disease (ESRD) or preexistent AF, patients enrolled in a health maintenance organization in 2006, and patients whose Medicare coverage changed were excluded.

The study period for assessing incident AF consisted of a 12-month baseline period and a maximum 2-year follow-up period. The baseline period was calendar year 2006, during which demographics, AF, CKD, and comorbid conditions were defined. Follow-up started on January 1, 2007, and ended at the earliest of the following events: incident AF, death, ESRD diagnosis, change in enrollment status, or December 31, 2008. All patients with incident AF during the follow-up period were included in the analysis for subsequent survival. During the baseline period of 12 months before the date of incident AF, CKD stage and comorbid conditions were ascertained. Follow-up began on the date of incident AF and ended at the earliest of the following events: death, ESRD diagnosis, or December 31, 2008. The maximum follow-up time was 2 years for patients with incident AF on January 1, 2007, and <2 years for patients with incident AF after January 1, 2007.

### Definition of Study Variables

Patients with preexisting AF were identified by at least one Part A inpatient, skilled nursing facility, or home health claim or two Part A outpatient or Part B claims on different days within a 12-month interval, carrying the *International Classification of Diseases, Ninth Revision, Clinical Modification* (*ICD-9-CM*) diagnosis code for AF (427.3x). Using the same algorithm for inpatient and outpatient claims, we subsequently identified patients with CKD according to the *ICD-9-CM* diagnosis codes listed in Table 1. The 5 stages of CKD were defined by *ICD-9-CM* codes 585.1–585.5, and code 585.9 was used for CKD stage unspecified/unknown. Comorbid conditions were identified similarly; Table 2 lists the relevant *ICD-9-CM* diagnosis codes. Date of incident AF was defined as the earliest date of a claim carrying the diagnosis code. Date of death was tracked directly from the 5% Medicare sample, and ESRD was defined via linkage to the United States Renal Data System ESRD registry.

**Table 1.** *ICD-9-CM* Codes Used to Define CKD

Disease	*ICD-9-CM* Diagnosis Codes
Renal tuberculosis	016.0
Syphilis of kidney	095.4
Kidney, except pelvis	189.0
Urinary organ, site unspecified	189.9
Kidney except pelvis	223.0
Kidney and ureter	236.91
Diabetes with renal manifestations	250.4
Renal glycosuria	271.4
Gouty nephropathy	274.1
Hemolytic-uremic syndrome	283.11
Hypertensive chronic kidney disease	403.x1
Hypertensive heart and chronic kidney disease	404.x2-3
Atherosclerosis of renal artery	440.1
Hyperplasia of renal artery	447.3
Hepatorenal syndrome	572.4
Acute glomerulonephritis	580
Nephrotic syndrome	581
Chronic glomerulonephritis	582
Glomerulonephritis or nephropathy NOS	583
Acute renal failure	584
Renal failure NOS	586
Renal sclerosis	587
Disorders resulting from impaired renal function	588
Hydronephrosis	591
Hypertension secondary to renal disease complicating pregnancy, childbirth, and the puerperium	642.1
Unspecified renal disease in pregnancy, without mention of hypertension	646.2
Polycystic kidney, unspecified type	753.12 to 753.17
Other specified cystic kidney disease	753.19
Obstructive defects of renal pelvis and ureter	753.2
Kidney, abnormal renal function test	794.4

CKD indicates chronic kidney disease; *ICD-9-CM, International Classification of Diseases, Ninth Revision, Clinical Modification;* and NOS, not otherwise specified.

**Table 2.** *ICD-9-CM* Codes Used to Define Comorbid Conditions

Disease	*ICD-9-CM* Diagnosis Codes	*ICD-9-CM* V Codes
MI	410; 412	
CHF	398.91; 422; 425; 428; 402.X1; 404.x1; 404.x3	V42.1
Other ASHD	411; 413; 414	V45.81; V45.82
Cardiac (other)	420 to 421; 423 to 424; 429; 785.0 to 785.3	V42.2; V43.3
CVA/TIA	430 to 438	
PVD	440 to 444; 447; 451 to 453; 557	
Anemia	280 to 285	
Cancer	140 to 172; 174 to 208; 230 to 231; 233 to 234	
COPD	491 to 494; 496; 510	
Diabetes	250; 357.2; 362.0x; 366.41	
GI disease	456.0 to 456.2; 530.7; 531 to 534; 569.84; 569.85; 578	
Hypertension	362.11; 401.x to 405.x; 437.2	
Liver disease	570; 571; 572.1; 572.4; 573.1 to 573.3	V42.7

ASHD indicates atherosclerotic heart disease; CHF, congestive heart failure; COPD, chronic obstructive pulmonary disease; CVA/TIA, cerebrovascular accident/transient ischemic attack; GI, gastrointestinal; *ICD-9-CM, International Classification of Diseases, Ninth Revision, Clinical Modification;* MI, myocardial infarction; and PVD, peripheral vascular disease.

### Statistical Analysis

Demographic characteristics and baseline comorbid conditions are presented as percentages and are compared among CKD stages with the χ^2^ test. Incidence of AF during the 2-year follow-up period and survival after incident AF with varying stages of CKD were estimated with the Kaplan-Meier method and were compared with the log-rank test. A multivariable Cox proportional-hazards model was used to assess the association between CKD stages and risk of incident AF and subsequent risk of death. The assumption of proportionality of the hazards was assessed through visual inspection. The covariates included in the model were baseline demographics and comorbid conditions. Possible effect modifiers of the association between CKD and incident AF and subsequent death were evaluated with adjustment for all other variables. Analyses were performed in SAS version 9.1 (SAS Institute, Cary, NC).

## Results

The 2006 Medicare cohort comprised 1 092 649 patients, of whom 55 962 (5.1%) had a diagnosis of CKD. Among the patients with CKD, 4952 (8.8%) had stages 1 and 2; 19 795 (35.3%), stages 3 to 5; and 31 215 (55.7%), unknown stage. Baseline characteristics are shown in Table 3. Compared with the non-CKD population, the CKD population included a significantly lower proportion of women (50% to 53% versus 60%, *P*<0.0001) and a higher proportion of black patients (12% to 15% versus 7%, *P*<0.0001). All comorbid conditions studied were significantly more prevalent in the CKD population and more prevalent with advancing CKD stages. In particular, prevalences of myocardial infarction, anemia, and congestive heart failure were almost 4-fold higher in the CKD than in the non-CKD population, and prevalences of other atherosclerotic heart disease, cerebrovascular accident/transient ischemic attack, diabetes, and hypertension were almost double (*P*<0.0001 for all comparisons).

**Table 3.** Baseline Patient Characteristics by CKD Stage Among Point-Prevalent Medicare Patients on December 31, 2006, Without AF or ESRD in 2006

	CKD Stage					
Characteristics	All	None	1 and 2	3 to 5	Unknown	*P*
Sample size, n	1 092 649	1 036 687	4952	19 795	31 215	
Age, y						<0.0001
66 to 69	23.3	23.7	17.5	14.3	14.6	
70 to 74	25.1	25.4	22.9	21.8	19.7	
75 to 79	21.3	21.3	22.9	24.0	22.2	
80 to 84	16.1	15.9	17.8	21.3	20.7	
≥85	14.2	13.8	19.0	18.7	22.8	
Sex						<0.0001
Men	40.8	40.4	50.1	48.9	46.6	
Women	59.2	59.6	49.9	51.1	53.4	
Race						<0.0001
White	87.6	87.8	79.6	81.3	83.4	
Black	7.6	7.4	14.9	13.4	11.5	
Other	4.8	4.8	5.6	5.3	5.1	
Comorbid conditions						
MI	2.3	1.9	6.8	8.1	10.1	<0.0001
Other ASHD	17.3	16.0	38.5	41.7	41.0	<0.0001
CHF	6.7	5.6	24.2	28.9	27.8	<0.0001
CVA/TIA	7.3	6.7	16.5	16.3	19.4	<0.0001
PAD	9.8	8.7	24.6	28.0	31.5	<0.0001
Other cardiac	9.2	8.4	21.5	22.2	23.7	<0.0001
Anemia	12.3	10.4	43.2	56.8	40.7	<0.0001
Cancer	9.6	9.1	15.2	15.7	21.9	<0.0001
COPD	11.2	10.5	21.1	20.4	24.6	<0.0001
Diabetes	20.4	19.0	48.2	49.5	44.3	<0.0001
GI bleeding	2.5	2.2	6.7	6.9	8.5	<0.0001
Hypertension	54.9	53.0	90.8	93.7	86.4	<0.0001
Liver disease	0.7	0.6	1.6	1.7	1.7	<0.0001

Unless otherwise indicated, values are percentages.

AF indicates atrial fibrillation; ASHD, atherosclerotic heart disease; CHF, congestive heart failure; COPD, chronic obstructive pulmonary disease; CVA/TIA, cerebrovascular accident/transient ischemic attack; CKD, chronic kidney disease; ESRD, end-stage renal disease; GI, gastrointestinal; MI, myocardial infarction; and PAD, peripheral arterial disease.

During the 2-year follow-up period, 79 135 patients were diagnosed with incident AF; incidence was higher in the CKD than in the non-CKD population. The estimated 2-year incidence of AF was 12.2% for patients with CKD stages 1 and 2, 14.4% for stages 3 to 5, and 13.4% for unknown stage, compared with 7.5% for patients without CKD (*P*<0.0001). The median follow-up time for incident AF was 24 months for patients without CKD and for each subgroup of patients with CKD. Table 4 presents the adjusted hazard ratios (HRs) of incident AF for patients with CKD by stage compared with patients without CKD. CKD stages 3 to 5 were modestly but significantly associated with incident AF (HR 1.13, 95% confidence interval [CI] 1.09 to 1.18). Increased age was strongly associated with a higher independent hazard of incident AF. Age 75 to 79 years was associated with a 2-fold higher risk and age ≥85 years with a 3.7-fold higher risk of incident AF. Female sex (HR 0.76, 95% CI 0.74 to 0.77) and black race (HR 0.64, 95% CI 0.62 to 0.66) were inversely associated with incident AF. With regard to comorbid conditions, congestive heart failure (HR 1.81, 95% CI 1.77 to 1.85) was most strongly associated with incident AF; other atherosclerotic heart disease, chronic obstructive pulmonary disease, and diabetes also were associated strongly with incident AF.

**Table 4.** Cox Proportional-Hazards Model Results for the Association Between CKD, by Stage, and Risk of Incident AF and Subsequent Survival

	Incident AF		Death After AF	
Characteristics	HR (95% CI)	*P*	HR (95% CI)	*P*
Sample size, n	1,092,649		79,135	
CKD status				
None	Reference		Reference	
Stages 1 and 2	1.02 (0.94 to 1.11)	0.6794	1.14 (1.00 to 1.30)	0.0426
Stages 3 to 5	1.13 (1.09 to 1.18)	<0.0001	1.27 (1.20 to 1.35)	<0.0001
Stage unknown	1.00 (0.97 to 1.04)	0.9089	1.29 (1.23 to 1.36)	<0.0001
Age, y				
66 to 69	Reference		Reference	
70 to 74	1.42 (1.38 to 1.46)	<0.0001	1.16 (1.07 to 1.25)	0.0003
75 to 79	2.01 (1.96 to 2.06)	<0.0001	1.38 (1.28 to 1.48)	<0.0001
80 to 84	2.66 (2.59 to 2.73)	<0.0001	1.89 (1.75 to 2.03)	<0.0001
≥85	3.66 (3.57 to 3.76)	<0.0001	3.31 (3.09 to 3.55)	<0.0001
Sex				
Men	Reference		Reference	
Women	0.76 (0.74 to 0.77)	<0.0001	0.96 (0.93 to 0.99)	0.0053
Race				
White	Reference		Reference	
Black	0.64 (0.62 to 0.66)	<0.0001	1.17 (1.10 to 1.24)	<0.0001
Other	0.63 (0.60 to 0.65)	<0.0001	1.03 (0.95 to 1.11)	0.503
Comorbid conditions				
MI	0.86 (0.83 to 0.89)	<0.0001	1.21 (1.14 to 1.27)	<0.0001
Other ASHD	1.33 (1.31 to 1.36)	<0.0001	0.80 (0.77 to 0.83)	<0.0001
CHF	1.81 (1.77 to 1.85)	<0.0001	1.52 (1.47 to 1.57)	<0.0001
CVA/TIA	1.07 (1.05 to 1.10)	<0.0001	1.20 (1.16 to 1.25)	<0.0001
PAD	1.25 (1.22 to 1.27)	<0.0001	1.14 (1.10 to 1.18)	<0.0001
Other cardiac	1.45 (1.42 to 1.48)	<0.0001	0.92 (0.88 to 0.95)	<0.0001
Anemia	1.05 (1.03 to 1.07)	<0.0001	1.43 (1.38 to 1.48)	<0.0001
Cancer	1.07 (1.04 to 1.09)	<0.0001	1.57 (1.52 to 1.63)	<0.0001
COPD	1.26 (1.24 to 1.29)	<0.0001	1.59 (1.54 to 1.65)	<0.0001
Diabetes	1.16 (1.14 to 1.18)	<0.0001	1.12 (1.08 to 1.16)	<0.0001
GI bleeding	0.94 (0.90 to 0.98)	0.0032	1.11 (1.05 to 1.18)	0.0005
Hypertension	1.09 (1.07 to 1.11)	<0.0001	0.83 (0.80 to 0.85)	<0.0001
Liver disease	1.11 (1.02 to 1.20)	0.0112	1.59 (1.42 to 1.79)	<0.0001

AF indicates atrial fibrillation; ASHD, atherosclerotic heart disease; CHF, congestive heart failure; CKD, chronic kidney disease; COPD, chronic obstructive pulmonary disease; CVA/TIA, cerebrovascular accident/transient ischemic attack; GI, gastrointestinal; HR, hazard ratio; MI, myocardial infarction; and PAD, peripheral arterial disease.

**Figure 1.**
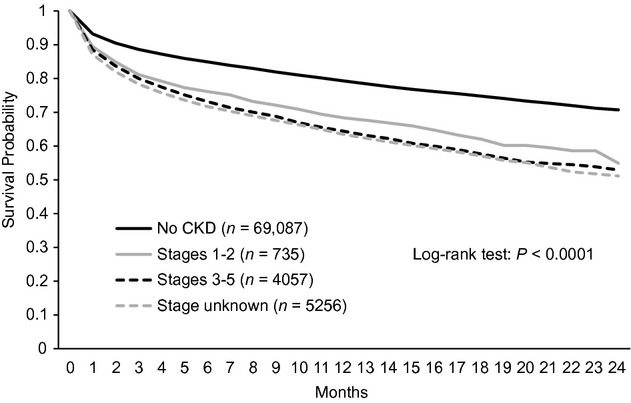
Unadjusted survival probability after incident AF by CKD stage in Medicare patients with AF in 2007–2008.

Of patients with incident AF during 2007–2008, 18 525 died during the follow-up period. Median follow-up times for the mortality rate analysis were 11.7, 9.4, 7.2, and 8.3 months for patients without CKD and patients with CKD stages 1 and 2, 3 to 5, and unknown stage, respectively.Figure 1 presents unadjusted estimates of survival after diagnosis of incident AF; survival was significantly reduced in patients with CKD compared with patients without CKD. At 12 months, survival was 79.3% for patients without CKD, 68.3% for patients with CKD stages 1 and 2, 64.4% for patients with stages 3 to 5, and 63.4% for patients with unknown stage. Corresponding values at 24 months were 70.7%, 54.9%, 52.9%, and 51.1%. *P* value for the log-rank test was <0.0001 for all comparisons.

The hazard of death was significantly higher for patients with CKD than for patients without CKD with incident AF, and the hazard was higher with advancing CKD stages. The hazards for stages 3 to 5 (HR 1.27, 95% CI 1.20 to 1.35) and unknown stage (HR 1.29, 95% CI 1.23 to 1.36) were higher than for stages 1 and 2 (HR 1.14, 95% CI 1.00 to 1.30) (Table 4). Increasing age also was associated with higher mortality hazards in patients with CKD with incident AF; HRs (95% CI) were 1.89 (1.75 to 2.03) for ages 80 to 84 years and 3.31 (3.09 to 3.55) for age ≥85 years. Comorbid conditions associated with the highest mortality hazard in patients with CKD and AF were congestive heart failure (HR 1.52, 95% CI 1.47 to 1.57), chronic obstructive pulmonary disease (HR 1.59, 95% CI 1.54 to 1.65), liver disease (HR 1.59, CI 1.42 to 1.79), and anemia (HR 1.43, 95% CI 1.38 to 1.48). Hypertension (HR 0.83, 95% CI 0.80 to 0.85) and atherosclerotic heart disease (HR 0.80 95% CI 0.77 to 0.83), when evaluated as comorbid conditions, were associated with reduced mortality rate in patients with CKD and AF. Hypertension was evaluated across CKD stages and various demographic subcategories; hypertension consistently showed a protective effect with regard to survival compared with absence of hypertension.

The effect modifications of several important covariates on the association between CKD and AF and subsequent death were evaluated with additional analysis. Age was the most important effect modifier (Table 5). The association between CKD stage and incident AF was strongest in the younger age group and was attenuated and reversed in the older age group. In the age group ≥85 years, patients with CKD had significantly lower risk of AF than patients without CKD. A similar trend was noted in the association of CKD stage and risk of death after incident AF. This association was highest in the group 66 to 69 years of age and progressively diminished until it became borderline significant in the group ≥85 years of age. On evaluation of race as a modifier, the risk of incident AF was similar in other CKD subgroups but was higher for black patients with unknown CKD stage (HR 1.26, CI 1.12 to 1.41). The risk of death associated with AF was significantly higher for white patients with CKD stages 3 to 5 (HR 1.26, CI 1.19 to 1.34) and unknown stage (HR 1.33, CI 1.26 to 1.41) but was not statistically significant for black patients in any CKD group (Table 6). The effect of CKD on incident AF was similar for men and women, but the effect on mortality rate after AF was stronger in women. The strength of the association between CKD and risk of AF was stronger in patients without hypertension but was attenuated and not statistically significant in patients with hypertension. Patients with CKD had a higher risk of death after AF than did patients without CKD, and the risk was elevated in the absence of hypertension.

**Table 5.** Association of CKD Stage and Risk of AF and Subsequent Death by Age

	Age in Years, HR (95% CI)					
	66 to 69	70 to 74	75 to 79	80 to 84	≥85	Overall, HR (95% CI)
Risk of AF						
No CKD	Reference	Reference	Reference	Reference	Reference	Reference
Stages 1 and 2	1.61 (1.26 to 2.06)	1.05 (0.85 to 1.30)	1.04 (0.87 to 1.24)	0.94 (0.78 to 1.13)	0.90 (0.77 to 1.06)	1.02 (0.94 to 1.11)
Stages 3 to 5	1.62 (1.41 to 1.85)	1.40 (1.27 to 1.54)	1.25 (1.16 to 1.36)	1.05 (0.97 to 1.14)	0.89 (0.82 to 0.96)	1.13 (1.09 to 1.18)
Stage unknown	1.44 (1.29 to 1.61)	1.28 (1.18 to 1.39)	1.09 (1.01 to 1.17)	0.96 (0.90 to 1.03)	0.80 (0.75 to 0.85)	1.00 (0.97 to 1.04)
Risk of death after AF						
No CKD	Reference	Reference	Reference	Reference	Reference	Reference
Stages 1 and 2	1.69 (1.02 to 2.83)	1.29 (0.87 to 1.90)	1.53 (1.13 to 2.07)	1.21 (0.92 to 1.60)	0.94 (0.77 to 1.15)	1.14 (1.00 to 1.30)
Stages 3 to 5	1.80 (1.40 to 2.32)	1.52 (1.29 to 1.78)	1.33 (1.17 to 1.52)	1.40 (1.25 to 1.56)	1.09 (1.00 to 1.19)	1.27 (1.20 to 1.35)
Stage unknown	1.69 (1.36 to 2.11)	1.84 (1.60 to 2.11)	1.66 (1.48 to 1.85)	1.22 (1.10 to 1.35)	1.09 (1.02 to 1.18)	1.29 (1.23 to 1.36)

AF indicates atrial fibrillation; CKD, chronic kidney disease.

**Table 6.** Association of CKD Stage and Risk of AF and Subsequent Death by Race, Sex, and Hypertension Status

	Race, HR (95% CI)				Sex, HR (95% CI)			Hypertension Status, HR (95% CI)		
	White	Black	Other	Overall	Men	Women	Overall	No	Yes	Overall
Risk of AF										
No CKD	Reference	Reference	Reference	Reference	Reference	Reference	Reference	Reference	Reference	Reference
Stages 1 and 2	1.02 (0.93 to 1.11)	0.98 (0.73 to 1.31)	1.20 (0.80 to 1.80)	1.02 (0.94 to 1.11)	0.98 (0.87 to 1.10)	1.07 (0.94 to 1.21)	1.02 (0.94 to 1.11)	1.70 (1.33 to 2.17)	0.97 (0.88 to 1.32)	1.02 (0.94 to 1.11)
Stages 3 to 5	1.14 (1.09 to 1.19)	1.15 (1.00 to 1.33)	1.01 (0.81 to 1.25)	1.13 (1.09 to 1.18)	1.11 (1.05 to 1.17)	1.16 (1.10 to 1.24)	1.13 (1.09 to 1.18)	1.64 (1.42 to 1.90)	1.10 (1.06 to 1.33)	1.13 (1.09 to 1.18)
Stage unknown	0.98 (0.94 to 1.02)	1.26 (1.12 to 1.41)	1.12 (0.94 to 1.33)	1.00 (0.97 to 1.04)	0.93 (0.88 to 0.97)	1.08 (1.03 to 1.14)	1.00 (0.97 to 1.04)	1.08 (0.98 to 1.20)	0.99 (0.96 to 1.34)	1.00 (0.97 to 1.04)
Risk of death after AF										
No CKD	Reference	Reference	Reference	Reference	Reference	Reference	Reference	Reference	Reference	Reference
Stages 1 and 2	1.16 (1.01 to 1.34)	1.05 (0.71 to 1.54)	0.85 (0.35 to 2.06)	1.14 (1.00 to 1.30)	1.01 (0.83 to 1.23)	1.28 (1.08 to 1.53)	1.14 (1.00 to 1.30)	1.08 (0.72 to 1.61)	1.15 (1.00 to 1.32)	1.14 (1.00 to 1.30)
Stages 3 to 5	1.26 (1.19 to 1.34)	1.03 (0.85 to 1.25)	2.15 (1.69 to 2.75)	1.27 (1.20 to 1.35)	1.20 (1.10 to 1.30)	1.34 (1.24 to 1.45)	1.27 (1.20 to 1.35)	1.49 (1.22 to 1.83)	1.25 (1.18 to 1.33)	1.27 (1.20 to 1.35)
Stage unknown	1.33 (1.26 to 1.41)	1.06 (0.90 to 1.25)	0.96 (0.73 to 1.25)	1.29 (1.23 to 1.36)	1.26 (1.17 to 1.35)	1.32 (1.24 to 1.42)	1.29 (1.23 to 1.36)	1.42 (1.24 to 1.62)	1.27 (1.21 to 1.34)	1.29 (1.23 to 1.36)

AF indicates atrial fibrillation; CKD, chronic kidney disease; and HR, hazard ratio.

## Discussion

In this large retrospective cohort study of Medicare patients, we found that advanced CKD is an independent risk factor for incident AF. Moreover, we demonstrated that incident AF in patients with advanced CKD is associated with a significantly increased 2-year mortality rate relative to patients without CKD. These observations are extremely relevant to the care of this elderly population and deserve close attention.

Data on the incidence of AF in patients with CKD are limited. Horio et al^12^ conducted a study of 1118 hypertensive patients from a single region in Japan and reported an increase in incident AF in hypertensive patients with CKD (adjusted HR 2.18, 95% CI 1.2 to 3.9). Similar to our findings, the relationship was statistically significant only among patients with advanced CKD (stages 4 and 5). Alonso et al^13^ reported a significant and progressively higher risk of incident AF with advancing CKD in 10 328 patients from varying ethnic backgrounds in the Atherosclerosis Risk In Communities Study. HRs reported with estimated glomerular filtration rate (eGFR) 15 to 29 and 30 to 59 mL/min per 1.73 m^2^ (equivalent to CKD stages 3 to 5) were 3.2 (95% CI 2.0 to 5.0) and 1.6 (95% CI 1.3 to 2.1), respectively.^13^ The observations from our study are complementary to those reported previously^12–13^ and validate the association between advanced CKD and incident AF in a large, elderly, and predominantly white population.

Several hypotheses have been offered to explain the relationship between CKD and increased rates of incident AF. Neurohormonal activation has been implicated in the progression of renal failure and the development of cardiovascular sequelae. Activation of the renin-angiotensin-aldosterone system in patients with CKD has been widely studied with regard to hypertension; angiotensin also has been shown to increase cardiac fibroblast proliferation and cardiac hypertrophy.^14–15^ Moreover, use of spironolactone has been associated with a reduced burden of AF, presumably related to mineralocorticoid receptor blockade and antifibrotic effects.^16^ In addition to the renin-angiotensin-aldosterone system, increased sympathetic activity in patients with CKD has been linked to increased serum concentration of androgenic hormones, and in turn, sympathetic activation has been proposed to contribute to arrhythmogenesis.^14^ In addition to neurohormonal activation, inflammation has been proposed as a contributor to increased incident AF in patients with CKD. Inflammatory markers such as C-reactive protein are elevated in patients with CKD and have been reported in association with incident AF. This observation is strengthened by findings of inflammatory changes in cardiac biopsy specimens from patients with AF.^17^ Furthermore, structural abnormalities, such as increasing left atrial diameter and left ventricular hypertrophy related to CKD, have been associated with increased risk of incident AF.^17^ L'Allier et al^18^ reported decreased incident AF among 10 926 hypertensive patients treated with angiotensin-converting enzyme inhibitors versus calcium channel blockers. These physiological changes could potentiate incident AF and therefore could have important implications pertaining to pharmacological management. In a large meta-analysis of several randomized controlled trials, Wang et al^19^ demonstrated that statin use is associated with significantly reduced risk of AF. Future studies should evaluate the role of inhibitors of the renin-angiotensin-aldosterone system and antiinflammatory medications (eg, statins) in reducing the burden of AF in this high-risk population.

Importantly, the present study provides compelling data indicating that patients with advancing CKD and incident AF have a significantly increased risk of death. A small number of studies have previously evaluated this association in patients with renal disease. Nakagawa et al^11^ published data from 387 Japanese patients without ESRD or renal transplant from a single center. The authors reported increased mortality rates among patients with nonvalvular AF and decreased eGFR. The HRs for all-cause death were 2.8 (95% CI 1.3 to 5.8) for patients with eGFR <60 mL/min per 1.73 m^2^ and CHADS_2_ score <2 and 6.9 (95% CI 3.5 to 13.5) for patients with eGFR <60 mL/min per 1.73 m^2^ and CHADS_2_ score ≥2.^11^ Although no distinction was made among patients with varying degrees of eGFR reduction in this selected population, the nearly 7-fold increase in the hazard for all-cause death in patients with higher thromboembolic risk scores deserves attention. Genovesi et al^20^ studied 476 patients with ESRD from 5 dialysis centers in one region of Italy and found that patients with AF had a 65% higher relative risk of death than patients without AF. Our study findings underscore the significant relation between the combination of CKD, especially more advanced CKD, and AF and increased mortality rate. Although higher mortality rates in patients with ESRD and AF have been described previously,^21^ this association has not been convincingly demonstrated in patients with CKD.

Multiple possible pathophysiological mechanisms could underlie these findings. One possible explanation for the higher mortality rate is a higher incidence of stroke in patients with CKD and AF. Hart et al^22^ reported a higher rate of ischemic stroke in patients with moderate CKD and AF from the Stroke Prevention in Atrial Fibrillation trials and showed that treatment with warfarin reduced this risk. However, management of AF in patients with CKD is significantly more complex because of the higher bleeding risk related to anticoagulation,^3,23^ which also could contribute to the increased observed mortality rate. Nakagawa et al^11^ found that patients with CKD, AF, and higher thomboembolic risk scores are at extremely high risk, but any potential contribution of anticoagulation to the increased mortality hazard in this population cannot be determined from this study. A more plausible possibility linking increased mortality rate with CKD and AF is that presence of AF reflects the composite effect of a spectrum of cardiovascular disease mechanisms leading to left atrial enlargement, including vascular stiffness/noncompliance (due to classic atherosclerosis and nonclassic risk factors) present in the metabolic milieu of CKD (ie, abnormal calcium and phosphate metabolism, left ventricular hypertrophy, and sympathetic overactivity). The genesis of AF in patients with CKD is intimately linked with the same mechanisms that predispose these patients to increased risk of cardiovascular death.

The importance of increasing age as an independent and powerful predictor of incident AF and of death after AF must be emphasized. Advancing age is associated with an increasing burden of cardiovascular comorbidity, including AF and worsening renal function. In a general Medicare population, Lakshminarayan et al^24^ demonstrated a progressively higher prevalence of AF with advancing age. For patients ≥85 years of age, prevalence of AF approached 12% in the Medicare 5% population. In a large population-based cohort, Baber et al^25^ showed progressively higher prevalence of AF with worsening stages of CKD; unadjusted rates of AF prevalence were 2.7% to 4.2% in patients with CKD stages 3 to 5. Importantly, however, the age-adjusted odds ratios for prevalent AF remained significantly higher in patients with advanced CKD than in patients without CKD.

Important covariates could modify the effect of CKD on incident AF and subsequent death. Interestingly, with increasing age, the association between CKD stage and risk of incident AF and subsequent survival weakened (Table 5). The importance of age as an effect modifier in relation to CKD and cardiovascular outcomes has been evaluated previously. In a large population of patients with CKD, Raymond et al^26^ showed that the risk of death was reduced with increasing age in individuals with similar degrees of CKD. Similarly, O'Hare et al^27^ found that the association of death and eGFR was stronger in younger patients and became progressively weaker with increasing age. This finding could represent survival bias from observational data; also, with advancing age, the association of CKD as a contributor to increased mortality rate could be diluted by higher background risk of death and comorbidity burden.^27^ In evaluating the improved survival rates of black versus white patients with CKD, Kovesdy et al^28^ explained these observations by noting differences in clinical characteristics and the fact that higher mortality rates of black patients in earlier CKD stages could lead to a survival bias for patients with fewer comorbid conditions in later CKD stages. Hypertension consistently has been shown to have a protective survival association in the elderly in CKD^27^ and non-CKD populations.^29^ In our study, we also found a beneficial survival association with hypertension, which could represent beneficial treatment effects of medications such as β-blockers^30^ that are used to treat hypertension.

Our study is limited in several important ways. The design is observational and retrospective. Although we demonstrate an association between advancing CKD and incident AF and subsequent death, these data do not demonstrate causality. Moreover, because of the large sample size, although some associations are statistically significant, their clinical significance might be relatively modest. Our data source is administrative; clinical data such as left ventricular ejection fraction, left atrial volume, functional class, and laboratory values (eg, serum creatinine) were not available. Additionally, unknown CKD stage lacks specificity, but its association with increased mortality rate and similar event rates suggest that it corresponds more closely to the lower end of the eGFR range, or stages 3 to 5 CKD,^3^ and previous studies have validated the high specificity of CKD diagnostic codes for presence of CKD.^31^

In conclusion, CKD is an independent risk factor for incident AF, and incident AF is significantly associated with increased mortality rate in Medicare patients with CKD. CKD and AF are coprevalent in the elderly, and our findings have significant implications in this burgeoning population. Future studies should further evaluate the pathophysiological mechanisms underlying these associations. Recognition of these relationships is an important first step toward development of pharmacological strategies to ameliorate the burden of these disease conditions and thereby reduce cardiovascular morbidity and mortality rates in the elderly.
